# RIT with Y90-Ibritumomab Tiuxetan in Follicular Non-Hodgkin Lymphoma: Evaluation of Recent Outcomes in a Single Institution

**DOI:** 10.1155/2012/412742

**Published:** 2012-09-25

**Authors:** Marcio Miguel Andrade Campos, Anel E. Montes Limón, Jose María Grasa, Paola Lievano, Teresa Baringo, Pilar Giraldo

**Affiliations:** ^1^Department of Haematology, Miguel Servet University Hospital, Zaragoza, Spain; ^2^Department of Nuclear Medicine, Miguel Servet University Hospital, Zaragoza, Spain; ^3^Instituto Aragonés de Ciencias de la Salud (I+CS), Zaragoza, Spain; ^4^Centro de Investigación Biomédica en Red de Enfermedades Raras (CIBERER). Zaragoza, Spain

## Abstract

*Background*. Based on historical data we reviewed our hospital clinical database to analyse our updated information and therapy outcomes of follicular non-Hodgkin lymphoma (F-NHL) patients treated with ^90^Y-Ibritumomab tiuxetan. *Patients and Methods*. Between 2005 and 2011, 56 F-NHL patients were included in a clinical protocol conducted by a multidisciplinary team and treated in the same centre. All patients received 0.3 or 0.4 mCi/kg IV (88%) of ^90^Y-IT; response evaluation was performed 12 weeks after. *Results*. M/F 44.6%/55.4%, mean age 61.45 years (30–85); ECOG 0-1 96.9%. According to FLIPI score, distribution were good: 58.5%, intermediate: 29.2%, and poor: 12.3%. Previous therapies: >2: 40% (26). ORR was 94.6% (53/56). CR: 85.7%; CR according to previous disease: relapsed disease: 90% (27/30), refractory disease: 42.85% (3/7), consolidation with CR: 92.85% (13/14), and consolidation with PR: 100% (5/5). Global PR and NR were 8.9% (5) and 5.3% (3), respectively. Mean OS 63.86 months with a mean follow-up time of 57 months (2–73). Mean TTP: 52.65 months (95% CI: 43.83–61.48). Median OS and TTP were not achieved. No hospital submissions or deaths were registered. *Conclusions*. This study confirms the safety and high efficacy of ^90^Y-IT in F-NHL patients, RIT in early stage of disease could improve outcomes.

## 1. Introduction

The beginning of immunotherapy with the approval of the anti-CD20-specific monoclonal antibody (Mo-Ab) rituximab by the Food and Drug Administration (FDA) in 1997, as part of the first-line treatment of non-Hodgkin B-cell lymphoma (NHL), marked an advance in response to treatment and overall survival in the lymphoproliferative disorders. The pivotal clinical trials of follicular non-Hodgkin lymphoma (F-NHL) that compared anthracycline-based combination chemotherapy with and without rituximab showed clear superiority, with reduced relative risk for treatment failure by 60% and significantly higher overall response rate (ORR) of 96% versus 90%, and prolonged duration of response [1]. These results were significant; however, cure cannot be guaranteed and still remains a therapeutic goal.

The particular radio sensitivity of lymphoid cells made radiation an attractive approach. Radioimmunotherapy (RIT) emerged as an option after Mo-Ab success, combining the selectivity of the anti-CD-20 immunotherapy with an attached radioisotope, making possible the delivery of radiation exactly within the tumor burden [2]. In 2002, the FDA approved the first radioimmunoconjugate (RIC) ^90^Y-ibritumomab (Zevalin), and soon thereafter a second RIC ^131^I-tositumomab (Bexxar) was approved, both efficient for the treatment of indolent or transformed, relapsed or refractory B-cell lymphoma [3].

^90^Y-ibritumomab tiuxetan (^90^Y-IT) is a monoclonal IgG1 *κ* anti-CD20 antibody attached to tiuxetan, a metal chelator who serves to stabilize the ytrium-90 radioisotope. It is a pure *β*-emitter that produces higher energy and longer path length radiation than ^131^I-tositumomab, making it advantageous for bulky, poorly vascularized and heterogeneous antigen expression tumors, and also feasible for outpatient treatment because of its selective beta isotope emissions [3]. 

In 2002, the phase III randomized study that compared ^90^Y-IT with rituximab immunotherapy in 143 patients with relapsed or refractory low-grade follicular or transformed CD20+ NHL showed significant ORR of 80% for the ^90^Y-IT group versus 56% for the rituximab group, and complete response (CR) rates of 30% versus 16%, respectively, with a median of duration of response (DR) of 14.2 months in ^90^Y-IT group versus 12.1 in control group, and time to progression (TTP) of 11.2 versus 10.1 months [4]. The proven result of ^90^Y-IT superiority gave rise to further investigation and new therapeutic horizons extending its use on first-line consolidation therapy. The results of the phase III randomized study of ^90^Y-IT as front-line consolidation, the first-line indolent trial (FIT) published in 2008, showed that ^90^Y-IT consolidation significantly prolonged median progression free survival (PFS) after a median of 3.5 years of observation, and converted 77% of partial response (PR) after induction into CR, resulting in a CR rate of 87% with low-associated toxicity [5, 6].

Since September 2005, therapy with ^90^Y-ibritumomab tiuxetan (Zevalin) has been available in the Miguel Servet University Hospital. Firstly applied only for relapsed and refractory F-NHL and after the approval included as part of consolidation therapy after first-line induction chemoimmunotherapy in patients with F-NHL. We have treated since then 65 patients, with an everyday clinical practice protocol, with same inclusion criteria and less subjective variations, reasons that make us believe our experience could be of great value. We present here the outcomes derived of its use. 

## 2. Patients and Methods

We have created a clinical protocol conducted by a multidisciplinary team formed by clinical hematologists, nuclear medicine physicians, radiopharmacy physicians, and nurses in the Miguel Servet University Hospital at Saragossa, Spain. It was started in September 2005 and established the use of ^90^Y-IT in patients with an excisional biopsy confirming the diagnosis of CD20+ F-NHL grade 1, 2, or 3 according to the revised classification system of the World Health Organization (WHO), and who had relapsed or refractory disease after at least a first-line combined chemotherapy, patients had been diagnosed and treated within the same center. In 2007, the therapeutic inclusion was extended as consolidation after first-line chemotherapy in patients with complete or partial response confirmed by PET/CTscan.

Before undergoing into RIT consideration, all patients were thoroughly examined and had a complete blood count (CBC) with leukocyte differential and platelet count, positron emission tomography PET/CTscan, and bone marrow aspiration, and biopsy was carried out. They were also tested for general blood chemistry (including serum creatinine, liver function tests, uric acid, and lactate dehydrogenase). 

Additional inclusion criteria applied to all cases were absolute neutrophil count (ANC) ≥ 1,500/*μ*L, absolute platelet count (APC) ≥ 100,000/*μ*L, and bone marrow total lymphocytes ≤ 25% by morphological counting, also serum bilirubin ≤ 2.0 mg/dL and serum creatinine ≤ 2.0 mg/dL. All patients were requested to sign an informed consent. 

**Figure 1:**
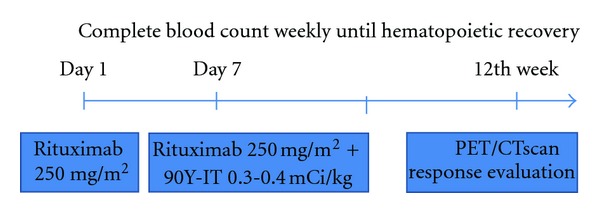
Therapeutic planning.

The therapeutic approach included the intravenous infusion of rituximab on day 1, at a dose of 250 mg/m^2^, to reduce nonmalignant binding with CD20+ circulating B cells and within the spleen, and the infusion of a second dose of rituximab at 250 mg/m^2^ on day 7, followed by weight-based dose of ^90^Y-IT administered as slow intravenous push over 10 minutes within the next 4 hours of rituximab infusion. The dose of ^90^Y-IT was 0.4 mCi ^90^Y/Kg in patients with platelet count >150, 000/*μ*L, and 0.3 mCi ^90^Y/Kg in patients with platelet count between 149,000 and 100,000/*μ*L. (Figure 1). FDA recommendations were taken into account.

A CBC was performed weekly in all patients within first 4 weeks after ^90^Y-IT infusion, or until if present, grade 3 or 4 cytopenia recovered, and then every two weeks until the CBC had normalized. Hematologic toxicity was defined according to WHO criteria. The target for transfusion was an APC less than 20,000/*μ*L in outpatients and hemoglobin level less than 8 g/dL or symptomatic anemia with poor tolerance. Prophylactic antibiotic therapy with levofloxacine was provided in all patients with an ANC less than 1,000/*μ*L until resolution, and those patients with grade 4 neutropenia received a single dose of PegG-CSF for recovery.

Response assessment was made 12 weeks after treatment; PET/CTscan was performed in all cases, and response criteria used were the same as the International Working Group (IWG). Subsequent follow-up evaluation also included CBC and physical examination every 3-4 months within the first 2 years after ^90^Y-IT therapy, and then every 6 months until relapse or death. A second PET/CTscan was performed at the first year of therapy, and a neck-chest and abdomino-pelvic CTscan was performed at the 5th year of ^90^Y-IT administration.

We assessed time to progression (TTP), overall response (OR) and OS in all patients. We also registered side effects, with special emphasis in myelotoxicity and emerging second neoplasms. OR was performed following the IWG criteria and classified as complete response (CR), partial response (PR), and no response (NR). TTP and OS were calculated from the date of ^90^Y-IT therapy until disease progression or death. All eligible patients were accepted by the clinical committee and included into the analysis. Patient data collection was cut-off at the last contact date. A database was created, and the statistical analysis of variables was performed with SPSS 15.0 program. Descriptive statistics, CIs and Statistical analysis of patient characteristics, response rate, and adverse events were descriptive. Analysis of TTP and OS were performed on intent to treat basis and were calculated by the Kaplan-Meier method with CIs.

## 3. Results

Between September 2005 and February 2012 a total of 65 patients who met the previously defined eligible criteria were treated with ^90^Y-IT in our hospital. 56 patients completed the follow-up and were considered into the analysis. Within demographic characteristics females were slightly more prevalent than males 55.4% (36) versus 44.6% (29). Mean age was 61.45 years (30–85), with overall good performance ECOG 0-1 in 96.9% of cases. The Ann Arbor stage distribution was IA 2 (3.1%); IIA 6 (9.2%); IIB 2 (3.1%); IIIA 5 (7.7%); IIIB 7 (10.7%); IVA 25 (38.5%); IVB 18 (27.7%). A total of 43 patients had bone marrow involvement at diagnosis. According to prognostic FLIPI score, the patient distribution was 58.5% (38) good, 29.2% (19) intermediate, and 12.3% (8) poor. 40% (26) patients had received more than two different combined chemotherapy schedules. According to status before ^90^Y-IT therapy, the patients were classified as consolidation after first-line therapy 22 (33.84%), relapsed with more than 12 months after previous therapies 31 (47.69%), refractory to rituximab schedules 7(10.77%), and partial response after first-line therapy 5 (7.70%). Main patients characteristics are detailed on Table 1.  Table 2 details previous schedules received by consolidation group. 

ORR was 94.6% (53/56). CR was achieved in 85.7% (48/56) patients. CR according to disease status before treatment is presented in Table 3. CR in relapsed disease 90% (27/30 valuable patients), in refractory disease 42.8% (3/7), in consolidation with CR after first-line therapy: 92.8% (13/14 valuable patients), and 100%(5/5) of patients that were in PR after first-line induction therapy converted to CR. PR was seen in 8.9% (5/56 valuable patients) and only 3 had no response. 16% (9/56) were non valuable patients, 8 were still within the first 12 weeks after ^90^Y-IT infusion, and 1 contact loss. According to bone marrow involvement 90.5% of patients obtained a complete response (38 of 42 patients). 

**Figure 2:**
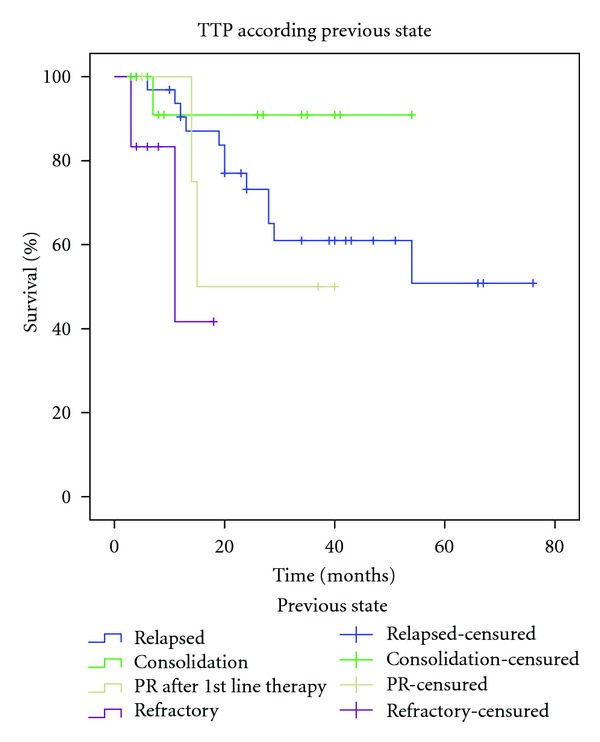
Time to progression (TTP) according to previous disease state before treatment.

**Figure 3:**
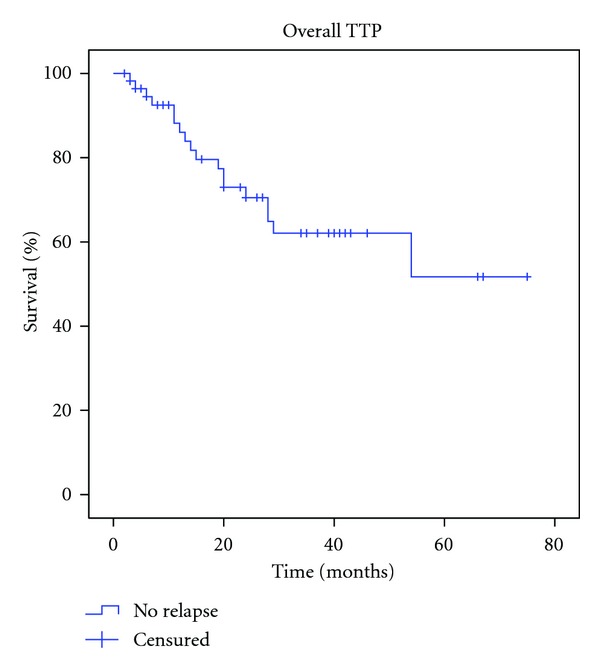
Mean global time to disease progression.

**Figure 4:**
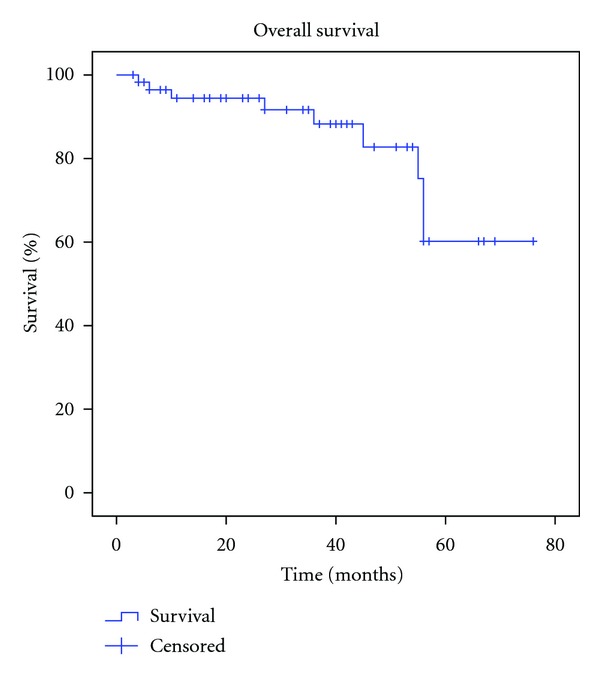
Mean estimated overall survival (OS).

Mean TTP in patients with relapsed disease was 52.56 months (CIs 95%: 42.31–62.81), with refractory disease 12.43 months (CIs 95%: 7.86–17), consolidation in CR after first-line induction therapy was 38.81 months (CIs 95%: 32.87–44.76), and consolidation in PR after first-line induction therapy was 27,25 months (CIs 95%: 14.75–39.75). By the end of the study 28.5% (16) have relapsed (Figure 2). Overall mean TTP was 52.65 months (SD+ 5.03, CIs 95%: 43.83–61.48) (Figure 3). The mean estimated OS was 63.86 months (CI 95%: 57.22–70.48) (Figure 4). Median estimated for OS and global TTP could not be calculated because of the good response of our patients at a median follow-up time of 57 months (range 2–73). 

We recorded side effects for safety assessment. Asthenia was the most frequent nonhematological adverse event presented in 50% of patients. Grade 3-4 thrombocytopenia was the most frequent hematological toxicity presented in 35.7% 20 patients, with a median time of onset around the 4th week after ^90^Y-IT infusion and spontaneous recovery around 8th week. 19% of patients developed grade 3-4 neutropenia within 4 weeks after ^90^Y-IT infusion, and spontaneous recovery occurred approximately 2 weeks later. 5 patients required red-cell transfusion, and 16 patients had platelet transfused. One patient developed grade 2 mucositis. None of the patients required hospitalization. Four patients had concomitant neoplasm at the time of treatment (colon, lung, breast, and prostate), and one patient developed prostate neoplasm four years after treatment at the age of 75. There was no treatment related mortality.

## 4. Discussion

^90^Y-IT therapy has proven to be efficient achieving complete and durable response rates including pretreated and rituximab refractory F-NHL patients, but there are not manylong term studiesusing this approach in relapsed/refractory follicular non-Hodgkin lymphoma patients andconsolidation [4, 5, 7–9].

The previous reported ORR on patients withrelapsed/refractory follicular non-Hodgkin lymphoma receiving ^90^Y-IT therapy is as high as 73%, and TTP as long as 3 or more years with even long-term responders reported (>5 years) [9]. The study published by Zinzani et al. in a single center with 57 patients evaluated the long-term outcomes of ^90^Y-IT and reported ORR of 93% at a median followup of 48 months and CR as high as 70% [10]. The median duration of response is estimated in 14.2 months [4] and more than 20 months [10] in two different publications. In our study the median duration of response was 27 months. In regard to consolidation therapy, the study FIT randomized phase III trial demonstrated a clear prolongation in TTP, higher response rates, and improvement of response in partial responders [5, 11], emerging the need of further investigation to establish a well defined therapeutic approach. 

The results obtained in our experience can be equalized with those previously reported elsewhere [10–15]. The CR achieved was 85.7%; overall TTP was around 52.65 months, with a mean estimated OS of 63.86 months and a mean follow-up time of 57 months (range 2–73), even in patients with bone marrow involvement at diagnosis. It seems that RIT with ^90^Y-IT in early stage of treatment induces higher and maintained CR, TTP, and OS (Figure 3). The evaluation of response to RIT, assessed by FDG-PET, has been reported as a main predictor of PFS, being PET/CTscan result post-RIT the only independent predictive factor [15].

^90^Y-IT was a good-tolerated and low-toxic therapy for the majority of patients, even for those heavily treated. The adverse events and myelotoxicity presented were expected, manageable, and transient, and there were none major side effects or related mortality, making it safe for outpatient administration and even suitable for elderly patients.

## 5. Conclusion

The addition of ^90^Y-IT into F-NHL treatment improves response and progression free survival of disease, both as part of relapsed or refractory disease, and as consolidation therapy after first-line induction treatment. Yet, still there is not unified criteria of the best approach and timing of RIT incorporation into treatment, and which chemotherapeutic associations can offer better response at less toxicity. This field needs further investigation in order to adequate RIT into schemes and take advantage of what seems a promising therapeutic tool. 

## Figures and Tables

**Table 1:** Patients characteristics (*N* = 65).

Characteristic	Number	%
Sex		
Male	29	55.4
Female	36	44.6
Age (years)		
Mean	61.45 years	
Range	30–85 years	
FLIPI		
Good	38	58.5
Intermediate	19	29.2
Poor	8	12.3
ECOG		
1-2		96.9
Prior treatment		
>2	26	4

**Table 2:** Schedules preconsolidation.

Treatment preconsolidation	Number of patients	%
R alone ×4	6	27.3
R-CHOP	13	59.1
R-EPOCH	1	4.5
R-COP	2	9.1

Total	22	100

R: Rituximab 375 mg/m^2^.

**Table 3:** Disease status before ^90^Y-IT therapy.

Status before treatment	Response to ^90^Y-IT treatment				Total
	CR	PR	NR	NV
Relapsed	27	1	2	1	31
Consolidation	13	1	0	8	22
Refractory	3	3	1	0	7
Partial response	5	0	0	0	5

Total	48	5	3	9	65

NHL-F: Non-Hodgkin follicular lymphoma; CR: complete response; PR: partial response; NR: no response; NV: non valuable.

## References

[1] Hiddemann W, Kneba M, Dreyling M (2005). Frontline therapy with rituximab added to the combination of cyclophosphamide, doxorubicin, vincristine, and prednisone (CHOP) significantly improves the outcome for patients with advanced-stage follicular lymphoma compared with therapy with CHOP alone: results of a prospective randomized study of the German Low-Grade Lymphoma Study Group. *Blood*.

[2] Press OW (1999). Radiolabeled antibody therapy of B-cell lymphomas. *Seminars in Oncology*.

[3] Illidge T, Morschhauser F (2011). Radioimmunotherapy in follicular lymphoma. *Best Practice & Research*.

[4] Witzig TE, Gordon LI, Cabanillas F (2002). Randomized controlled trial of yttrium-90-labeled ibritumomab tiuxetan radioimmunotherapy versus rituximab immunotherapy for patients with relapsed or refractory low-grade, follicular, or transformed B-cell non-Hodgkin’s lymphoma. *Journal of Clinical Oncology*.

[5] Morschhauser F, Radford J, van Hoof A (2008). Phase III trial of consolidation therapy with yttrium-90-ibritumomab tiuxetan compared with no additional therapy after first remission in advanced follicular lymphoma. *Journal of Clinical Oncology*.

[6] Morschhauser F, Dreyling M, Rohatiner A, Hagemeister F, Bischof Delaloye A (2009). Rationale for consolidation to improve progression-free survival in patients with non-Hodgkin’s lymphoma: a review of the evidence. *The Oncologist*.

[7] Emmanouilides C (2009). Review of ^90^Y-ibritumomab tiuxetan as frist-line consolidation radio-immunotherapy for B-cell follicular non-Hodgkin’s lymphoma. *Cancer Management and Research*.

[8] Hainsworth J, Spigel D, Markus T (2009). Rituximab plus short-duration chemotherapy followed by Yttrium-90 ibritumomab tiuxetan as first-line treatment for patients with follicular non-hodgkin lymphoma: a phase II trial of the Sarah Cannon oncology research consortium. *Clinical Lymphoma & Myeloma*.

[9] Gordon LI, Molina A, Witzig T (2004). Durable responses after ibritumomab tiuxetan radioimmunotherapy for CD20^+^ B-cell lymphoma: long-term follow-up of a phase 1/2 study. *Blood*.

[10] Zinzani PL, Gandolfi L, Stefoni V (2010). Yttrium-90 ibritumomab tiuxetan as a single agent in patients with pretreated B-cell lymphoma: evaluation of the long-term outcome. *Clinical Lymphoma, Myeloma and Leukemia*.

[11] Illidge TM (2010). Radioimmunotherapy of lymphoma: a treatment approach ahead of its time or past its sell-by date?. *Journal of Clinical Oncology*.

[12] Witzig TE, White CA, Wiseman GA (1999). Phase I/II trial of IDEC-Y2B8 radioimmunotherapy for treatment of relapsed or refractory CD20^+^ B-cell non-Hodgkin’s lymphoma. *Journal of Clinical Oncology*.

[13] Witzig TE, Molina A, Gordon LI (2007). Long-term responses in patients with recurring or refractory B-cell non-Hodgkin lymphoma treated with yttrium 90 ibritumomab tiuxetan. *Cancer*.

[14] Witzig TE, Flinn IW, Gordon LI (2002). Treatment with ibritumomab tiuxetan radioimmunotherapy in patients with rituximab-refractory follicular non-Hodgkin’s lymphoma. *Journal of Clinical Oncology*.

[15] Weigert O, Illidge T, Hiddemann W, Dreyling M (2006). Recommendations for the use of Yttrium-90 ibritumomab tiuxetan in malignant lymphoma. *Cancer*.

